# Advocacy Curricula in Graduate Medical Education: an Updated Systematic Review from 2017 to 2022

**DOI:** 10.1007/s11606-023-08244-x

**Published:** 2023-06-20

**Authors:** Nupur Agrawal, Jessica Lucier, Rikke Ogawa, Abigail Arons

**Affiliations:** 1 Division of Internal Medicine and Pediatrics, Department of Internal Medicine, University of California Los Angeles David Geffen School of Medicine, Los Angeles, CA USA; 2 Palliative Care Program, Division of General Internal Medicine & Health Services Research, Department of Internal Medicine, University of California Los Angeles David Geffen School of Medicine, Los Angeles, CA USA; 3 UCI Libraries, University of California Irvine, Irvine, CA USA; 4 Division of General Internal Medicine, Department of Internal Medicine, Department of Pediatrics, University of California San Francisco, San Francisco, CA USA

## Abstract

**Background:**

Advocacy is an integral component of a physician’s professional responsibilities, yet efforts to teach advocacy skills in a systematic and comprehensive manner have been inconsistent and challenging. There is currently no consensus on the tools and content that should be included in advocacy curricula for graduate medical trainees.

**Objective:**

To conduct a systematic review of recently published GME advocacy curricula and delineate foundational concepts and topics in advocacy education that are pertinent to trainees across specialties and career paths.

**Methods:**

We conducted an updated systematic review based off Howell et al. (J Gen Intern Med 34(11):2592–2601, 2019) to identify articles published between September 2017 and March 2022 that described GME advocacy curricula developed in the USA and Canada. Searches of grey literature were used to find citations potentially missed by the search strategy. Articles were independently reviewed by two authors to identify those meeting our inclusion and exclusion criteria; a third author resolved discrepancies. Three reviewers used a web-based interface to extract curricular details from the final selection of articles. Two reviewers conducted a detailed analysis of recurring themes in curricular design and implementation.

**Results:**

Of 867 articles reviewed, 26 articles, describing 31 unique curricula, met inclusion and exclusion criteria. The majority (84%) represented Internal Medicine, Family Medicine, Pediatrics, and Psychiatry programs. The most common learning methods included experiential learning, didactics, and project-based work. Most covered community partnerships (58%) and legislative advocacy (58%) as advocacy tools and social determinants of health (58%) as an educational topic. Evaluation results were inconsistently reported. Analysis of recurring themes showed that advocacy curricula benefit from an overarching culture supportive of advocacy education and should ideally be learner-centric, educator-friendly, and action-oriented.

**Discussion:**

Combining core features of advocacy curricula identified in prior publications with our findings, we propose an integrative framework to guide design and implementation of advocacy curricula for GME trainees. Additional research is needed to build expert consensus and ultimately develop model curricula for disseminated use.

**Supplementary Information:**

The online version contains supplementary material available at 10.1007/s11606-023-08244-x.

## INTRODUCTION

Advocacy is a key component of the modern physician’s professional responsibilities according to many influential medical organizations, including the Accreditation Council for Graduate Medical Education (ACGME)^1]^, American Academy of Pediatrics (AAP)^2^, American Board of Internal Medicine (ABIM)^3^, American College of Physicians (ACP)^3^, American Medical Association (AMA)^4, 5^, American Psychiatric Association (APA)^6^, and Royal College of Physicians and Surgeons of Canada^7–22^. ACGME common program requirements for residents and fellows across specialties acknowledge education on social determinants of health (SDOH) and health disparities^16^^,^^23, 24^ as essential; advocacy education is included but in a very limited scope as it pertains to direct patient care and patient care systems.^25^ The CanMEDS framework used in Canada more explicitly identifies *health advocate* as one of seven core abilities physicians must demonstrate to expertly care for patients^15^^,^^22^.

Graduate medical trainees across specialties^13, 14^^,^^21^ have demonstrated interest in learning about advocacy and developing practical advocacy skills.^13^ As the last intensive didactic opportunity prior to independent medical practice, the graduate medical education (GME) years present a prime opportunity to teach advocacy. Data suggests that meaningful engagement in advocacy can reinforce physician identity and the choice to practice medicine^8–11^^,^^26^, provide an outlet for stress management^27^, mitigate burnout^7–9^^,^^26^^,^^28^, and support professional development^29^. Despite increasing recognition of its importance, advocacy education remains elusive and challenging. Residents and fellows face significant time constraints due to extensive direct patient care responsibilities, and some trainees may even consider advocacy-related activities to be burdensome or unrealistic given competing demands^21^. Advocacy is also difficult to teach and assess^30^. Lack of formal^7, 8^^,^^11^^,^^19^^,^^21^, explicit^9^^,^^31^, and consistent^11^^,^^16^^,^^32^ training leads to inadequate preparation^13^^,^^28^^,^^33^ of trainees and negatively impacts patient care^13^. Perhaps most challenging for educators in this space has been the lack of formal curricula and guidelines to facilitate teaching efforts^15–17^^,^^19^.

In 2019, Howell et al. published the first systematic review analyzing methodologies, structure, content, facilitating factors, and barriers across GME advocacy curricula^34^. They analyzed 38 articles published through September 2017 and found that the most common tools covered in advocacy curricula included health policy and legislative advocacy, persuasive communication (media advocacy, op-ed writing, public speaking), grassroots advocacy, community partnership, and research-based advocacy. They concluded that advocacy education can “benefit from continued development of standardized goals, content, and outcome measures to better correlate with stated educational objectives.”.^34^

Since this initial review 5 years ago, a spotlight on structural inequities and resultant health disparities, laid bare by the COVID-19 pandemic, has pushed physician advocacy to the forefront of national conversation^35–37^, and many programs have published their GME advocacy curricula, sharing creative solutions to barriers as well as lessons learned in curricular implementation. Given the substantial effort and expertise required to develop curricula de novo, several authors have called for developing and disseminating advocacy curricula that can be adapted across GME programs and specialties^16^^,^^21^. We hypothesized that this new wave of articles represents a critical mass sufficient for investigating any significant changes in advocacy education in the last 5 years and analyzing common themes and program structures that could serve as a basis for an eventual model curriculum. For these reasons, we conducted an updated systematic review utilizing a search strategy similar to that created by Howell et al. to evaluate GME advocacy curricula published from September 2017 to March 2022. Building on the foundational concepts presented in the Howell review, our work aimed to delineate a comprehensive landscape of GME advocacy curricula and provide new insights into the common components (e.g., logistics, tools, content, evaluation methods), key barriers, and best practices that can guide development of model advocacy curricula for disseminated use across GME programs and specialties.

## METHODS

A medical librarian (RO) conducted systematic literature searches in PubMed (NLM), Embase (Embase.com), PsycINFO (ProQuest), and Educational Resources Information Center (ERIC via ProQuest) databases (Appendix). Search strategies for PubMed, Embase, and PsycINFO were replicated using those published in the prior study^34^. The original documentation did not reveal a replicable ERIC strategy, so a new strategy was designed, mapping closely to the original intent of the PsycINFO database strategy. Citations were included from September 1, 2017, to March 4, 2022. One author (AA) searched MedEdPORTAL (see the Appendix for search strategy) to identify additional curricular content published September 2017 forward. As in the prior study, search concepts included graduate medical education, curriculum, advocacy, community engagement, human rights, social justice, lobbying, vulnerable populations, and poverty. Deduplication algorithms in EndNote™^38^ were run against all citations.

Inclusion and exclusion criteria were determined using the prior study’s criteria as a guide: English language manuscripts published between September 1, 2017 and March 4, 2022, describing graduate medical educational curricula in US and Canadian programs and explicitly discussing concepts related to advocacy training. Articles were excluded if the topic had narrow educational scope (i.e., limited to only clinic-based quality improvement and population health, only individual patient advocacy, or describing a single community resource), described programs outside the USA and Canada, published as an abstract only, or published in a language other than English. Similar to the prior study, we used the Earnest et al. definition of advocacy: “action by a physician to promote social, economic, educational, and political changes that ameliorate the suffering and threats to human health and well-being.”.^5^

Two reviewers (AA, JL) independently screened titles and abstracts of all citations identified in the initial search for inclusion and exclusion with disputes resolved by a third reviewer (NA). Two reviewers (JL, NA) then hand searched the bibliographies of included articles for potential articles not previously identified. One reviewer (NA) also reviewed all articles citing the original Howell study, which revealed additional articles from Web of Science and PubMed. Duplicate articles were identified and removed. One reviewer (NA) read the abstracts for all articles identified via these additional search methods. No automation tools, other than EndNote™, were used during any step of the search process, and the strategy was not registered with PROSPERO since it was derived directly from the prior study.

All three reviewers (AA, JL, NA) read the final set of selected articles; each reviewer read the articles in a different order to prevent fatigue-related bias toward the end of the article set (AA read in alphabetical order by author last name; JL read in reverse alphabetical order by author last name; NA read in random order). Google Forms™, a web-based interface, was used to extract curricular details. General information about the curricula (e.g., country, institution, specialty, requirement, duration, teaching methods) was gathered as done in the preceding systematic review. We also extracted details of skills-based “advocacy tools” (e.g., legislative advocacy, community partnership, etc) and looked at knowledge-based “content areas” (e.g., SDOH, health equity and racial justice, quality improvement as a systemic policy rather than a process, and major health legislation). Data was gathered on evaluation methods beyond the original review article.

Once data was comprehensively extracted from each article, two reviewers (NA, AA) conducted a detailed synthesis. Original articles were reviewed again, and discrepancies were discussed to resolve inconsistencies in analysis. Additional notes from each reviewer beyond evaluation of standard curricular components were summarized. A summary statement for each article was generated and analyzed for recurring themes in curricular design and implementation.

## RESULTS

**Figure 1:**
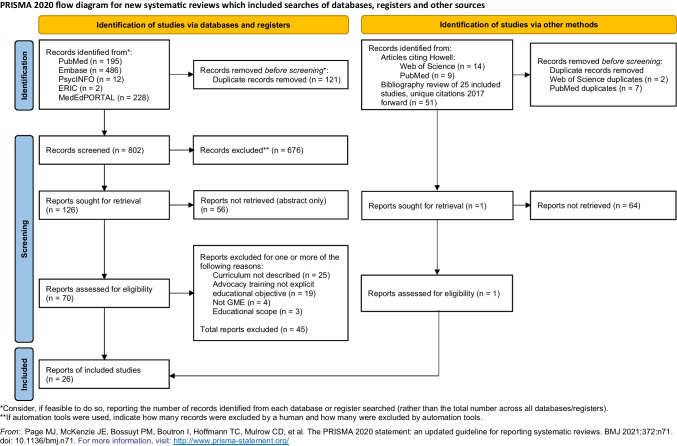
PRISMA diagram.

Our initial search produced 802 citations that were evaluated for inclusion and exclusion, with 126 articles identified for full text review. Twenty-five articles ultimately met all inclusion and exclusion criteria. A hand search of the bibliographies of these articles and a review of all articles citing the initial Howell study produced an additional 65 citations of which one met all inclusion and exclusion criteria (Fig. 1).

The final set of 26 included articles represented 31 total GME curricula, as two articles (Vance and Kennedy) each detailed the same set of seven unique Psychiatry curricula. Table 1 summarizes the content and logistical structure for each included curriculum. US-based programs accounted for the majority of curricula (30 of 31); only one was from Canada. Curricula were most commonly found in Internal Medicine, Family Medicine, Pediatrics, and Psychiatry programs with only 16% representing other specialties, including Obstetrics and Gynecology, Physical Medicine and Rehabilitation, Preventive Medicine, and various surgical subspecialties.

**Table 1:** Summary of Included Curricula

First author year	Institution	Specialty	Required/elective	Protected time	Logistics	Summary of curriculum
Andrews 2019^7^	Tulane	Internal Medicine	Elective	Yes	3-year track. Monthly evening seminars, annual retreat; optional 4 weeks of elective time per year to focus on advocacy project	Advocacy and leadership track. Residents complete a “portfolio” of advocacy experiences, including seminars, retreat, leadership and writing workshops, mentored longitudinal project, and broadly defined “advocacy actions.”
Bromage 2019^32^	Yale	Psychiatry	Required	Yes	3 sessions for PGY-2 s. Community-based evening, neighborhood tour day, and presentation session	Structural competency initiative. Art gallery night, immersive neighborhood tours with peer advocates and community leaders, resident presentation session critiqued by panel of community leaders
Campbell 2020^12^	Northwestern University	Internal Medicine	Required	Yes	3 75-min evening modules over 3 months	SDOH curriculum, article does not provide details on curricular content
Emery 2022^11^	Cambridge Health Alliance (CHA)	13 Boston—programs, specialty not specified	Required for 1 program, optional for all others	No	3 h within a larger 1-day health equity event	Workshop on “public narrative” tool for community organizing. Interns develop their own story with coaches, and reflect on using these skills to support health equity during residency
Gimpel 2017^39^	University of Texas Southwestern	Family Medicine	Elective	Yes	3-year track: 4-week block each year, plus 3 elective months in PGY-3 year	CARE track. 3-year mentored academic research project, annual community medicine rotation and 3–4 half days per year at underserved clinic, Opportunities for MPH course work
Goss 2020^8^	Montefiore	Internal Medicine: Primary Care and Social Medicine residency	Unclear	Yes	Multiple seminars over 3 years	Liberation medicine curriculum. Sessions led by faculty and community leaders to introduce and define liberation medicine and apply to policy issues. Includes mentorship and advocacy writing series
Hirsch 2017^40^	University of North Carolina	PM&R	Required	Yes	1-h debate with preparation and follow-up	Panel debate on proposed Affordable Care Act changes. Residents did mentored preparation of slides/summaries, to represent major stakeholder groups. Audience of faculty and staff spectators
Jones 2018^29^	University of Utah	Family Medicine	Required	No	1 blog post per resident per year, over 3 years	Family Medicine Vital Signs blog. Each resident and faculty member contributes at least one blog post per year on an advocacy topic of their choosing, supported by editorial board
Khera 2022^16^	Jersey City Medical Center	Internal Medicine	Required	Yes	10-h-long didactic sessions over 1 year, once every 5 weeks in 5 + 1 system	10-module curriculum on policy and community-based tools. Roundtable conversations reviewing tools and in-depth policy topics, clinic-based population health exercises, annual projects, senior resident mentorship of interns
Knox 2018^26^	Aurora Health Care (WI)	Family Medicine	Required	Yes	10 h during intern orientation, 1 month blocks in PGY1 and 2 years; Additional elective time in PGY2/3	Community Health, Advocacy, and Managing Populations (CHAMP) curriculum. Core principles of community health and SDOH, population health management, elective project and opportunities for unique clinical environment
Krishnaswami 2018^28^	University of Texas Rio Grande Valley	Preventative Medicine	Required	Yes	2-year dedicated residency	Community-Engaged Lifestyle Medicine residency. Clinical activities (partnerships with promotoras, home visits), didactics (all residents earn MPH), immersion experiences (5-month rotations doing public health and policy work), mentored research projects
Lax 2019^13^	Children’s Hospital of Montefiore	Pediatrics	Required	Yes	6 workshops and lobby day, over 9 months	Problem-based learning series plus speakers. Teaches 3-tier model of advocacy (patient, community, legislature), clinical skills (SDOH screening, community referrals), and advocacy topics (government benefits, educational advocacy, legal partnerships). Lobby day
Majeed 2020^20^	Eastern Virginia Medical School	Pediatrics	Elective	Yes	4 lectures, 3 workshops over 1 year during noon conference	Workshops facilitated by local faculty and advocacy leaders. Pair training on a tool (legislative visits, op eds, negotiation) with a specific child health policy issue. Residents prepare an advocacy action plan that they bring to a state lobby day to discuss
Michelson 2019^41^	Boston Combined Pediatric Residency Program	Pediatrics	Required	Yes	12-week longitudinal integrated block during intern year	Integrated rotation combining advocacy, developmental-behavioral pediatrics, and emergency medicine. Advocacy portions include seminar series, weekend and evening advocacy activities in the community, self-directed advocacy project
Neff 2020^9^	Developed at UCSF, run at multiple local institutions	Internal Medicine; Psychiatry; Family Medicine	Unclear	No	3–4-h workshop, implemented in 32 distinct instances between 2015 and 2017	Interprofessional structural competency workshop. Led by 2–3 facilitators from diverse backgrounds (MD, RN, sociology, etc.) who have undergone a training, detailed in the article
Oldfield 2018^31^	Johns Hopkins	Internal Medicine; Med Peds	Required	Yes	Residency track with 4-h academic half days every 2 weeks, and additional electives	Urban health residency track, with advocacy curriculum incorporated. Academic half days include journal club, leadership, and stakeholder engagement training. Optional book club, and option for part-time master’s degree
Pak-Gorstein 2018^18^	Seattle Children’s	Pediatrics	Elective	Yes	4 blocks during PGY2-3 years (2 1-month blocks PGY2, 2-month block PGY3)	Resident Education in Advocacy and Child Health (REACH) program. Didactics on community engagement, advocacy training, clinical perspectives on resource-limited settings. Personal career development plan. PGY-3 immersion
Piel 2018^19^	University of Washington	Psychiatry	Elective	Yes	6-month 1/2-day per week rotation, consisting of a 12-week evening course, and a 12-week research project	Forensic psychiatry. Psychiatry and the law course cross-listed with law school. Extensive, mentored project, either legal research, or participation in the legislative process (drafting legislation, working with advocacy organizations, giving testimony, etc.)
Sieplinga 2021^10^	Michigan State University	Pediatrics	Required	Yes	28 days as either 1st or 2nd block of PGY-1 year	Integrated Community Health and Child Advocacy (ICHCA) curriculum. Interns see patients in the morning and then have advocacy time in the afternoon where they work with community agency leaders, and do asset mapping project
Teran 2020^17^	University of Texas Austin, San Antonio Health Science Center, UTSW	Pediatrics	Required	No	5-min sessions at existing teaching conferences, 5 times over 5 months	Brief advocacy alert presentations designed and delivered by residents. Cover child health advocacy topics and suggested voluntary actions (e.g., calling or send email to state legislators), with follow-up materials
Traba 2021^27^	10 New Jersey Pediatric residency programs	Pediatrics	Unclear	No	9 webinars over 3 months	Virtual House Call webinar series. Speakers including pediatricians, community leaders, and law professionals, responding to COVID-related topics in real time.*
Vance 2020;^14^ Kennedy 2018^21^	Harvard/Massachusetts General Hospital/McLean Hospital	Psychiatry	Required	Yes	1 h per year, plus a 1-h lecture during PGY-2 and a 3-h panel during PGY-3	3 lecture series. Advocacy fundamentals series; Racism as SDOH series; Structural competency series. Resident competition for community project funding
	Hennepin County Medical Center	Psychiatry	Required	Yes	PGY2 seminar series and group project; additional electives	Resident-led seminars on “Social Determinants of Mental Health” (APA publication). PGY2s do joint annual service project. Optional legislative advocacy retreat day for all residents, elective for administrative shadowing
	UCSF	Psychiatry	Unclear	Yes	7 h over 2 years	Didactics reviewing frameworks for advocacy, policy and stakeholder engagement, structural competency, writing for a public audience. Longitudinal advocacy project with faculty mentorship
	University of Illinois, Peoria	Psychiatry	Required	Yes	2 lectures (1.5–2 h) and 1 outside speaker over 4 years; advocacy day in PGY-3 year	Lectures on basics of legislative process, mental health advocacy, with guest speakers. Residents learn to prepare a 1-page memo. PGY-3 advocacy day
	University of Michigan	Psychiatry	Required	Yes	Didactics over 4 years with additional elective	Required didactic curriculum over 4 years (PGY1/2 psychiatry in social context; PGY3/4 healthcare systems, financing, legal regulation of psych practice across states). Additional electives
	University of Texas Southwestern	Psychiatry	Required	Yes	2 h of PGY-1 lecture/discussion sessions plus workshop held once every 4 years	Workshop with 1 h didactic and 3 h of panel and breakout groups, topics chosen by resident interest. Mental Health Day at State Capitol. PGY1s get components of the workshop in 2 h of lectures/discussion sessions
	Yale	Psychiatry	Required	Yes	4.5 h over 3 sessions in PGY-2 core curriculum, with additional electives for PGY-3/4 and fellows	PGY2 core curriculum on social justice and health inequity, with 1 didactic session on community advocacy, 2 sessions on legislative advocacy. Electives include legislative visits, preparing testimony, and guest lectures
Webber 2018^42^	University of Wisconsin-Madison	Pediatrics; Family Medicine	Elective	Yes	5-day curriculum as a part of Fundamentals of Global Health Course	Local Global Health sessions. Specific focus on Latinx community, use Asset-Based Community Development (ABCD) framework, applied to local community via exercises in partnership with community leaders
Whetstone 2018^33^	UCSF	Ob/Gyn	Required	Yes	Curriculum of required didactics (lectures, grand rounds), PGY-2 clinical experience, with optional 4-week global health component in Uganda during PGY-3	EMPOWUR (Educating, Mentoring, and Preparing Ob/Gyns to care for Women in Under-Resourced communities) curriculum. Didactics on SDOH. Disparities, and advocacy training. Direct care in underserved communities block. Role modeling thread with guest speakers. Uganda experience with focus on capacity building
Ying 2019^15^	University of Ottawa	Surgery (multiple specialties)	Required	Yes	3 h of protected time during a weekly academic half day in surgical foundations curriculum	Community outreach initiative. Residents complete advocacy project, encouraged to be related to surgical specialty, in groups or individually, with peer presentation at conclusion

*This was initially a cross-residency collaborative with faculty and residents to build community partnerships with Family Success Centers (FSCs) and develop a core advocacy curriculum for all sites; conducted needs assessment and obtained implementation grant. However, given roll out during April–June 2020, the residencies pivoted

### Curricular Methods, Tools, and Content

Curricular details on teaching methods, skills-based advocacy tools, and knowledge-based advocacy and policy content areas are shown in Table 2. Table 3 summarizes frequency of specific characteristics across curricula.

**Table 2:** Educational Methods and Content of Included Advocacy Curricula

First author, year		Teaching methods						Advocacy tools						Content areas						
		Experiential learning	Small group/seminar^1^	Lecture^1^	Independent project	Group project	Required reading	Legislative advocacy	Community partnership	Advocacy writing	Public speaking	Research-based advocacy	Media relations	SDOH	Health equity/racial justice	Healthcare finance	Quality improvement	Major health legislation	Structural competency	Global health
Andrews 2019		●	●		●	●		●		●			●	●						
Bromage 2019		●	●			●	●		●					●	●				●	
Campbell 2020			●	●										●	●					
Emery 2022			●	●					●											
Gimpel 2017		●		●	●				●			●								
Goss 2020				●					●	●				●	●					●
Hirsch 2017							●				●							●		
Jones 2018		●			●					●										
Khera 2022		●	●	●					●					●		●	●	●		
Knox 2018		●		●	●			●	●	●		●		●	●					
Krishnaswami 2018		●	●	●	●		●	●	●	●		●		●	●	●	●			
Lax 2019		●	●				●	●		●				●						
Majeed 2020			●	●			●	●		●				●						
Michelson 2019		●																		
Neff 2020			●	●					●					●	●				●	
Oldfield 2018		●	●	●				●	●					●	●					
Pak-Gorstein 2018		●	●	●				●	●	●	●	●	●	●	●					●
Piel 2018			●	●	●		●	●		●		●								
Sieplinga 2021		●		●				●	●	●			●	●			●			
Teran 2020								●												
Traba 2021		●							●											
Vance 2020; Kennedy 2018	Harvard	●		●				●	●					●	●				●	
	Hennepin	●	●			●	●	●	●					●						
	UCSF					●		●		●									●	
	U. Illinois	●		●				●		●										
	U. Michigan	●		●				●						●		●				
	UTSW		●	●				●												
	Yale			●		●		●	●	●	●		●	●	●			●		
Webber 2018		●		●		●	●		●					●	●					●
Whetstone 2018		●	●	●		●		●	●									●		●
Ying 2019		●							●											

^1^Small group/seminar and lecture are both considered to be didactic in nature

**Table 3:** Descriptive Statistics of Included Advocacy Curricula

	*n* (%, with total *n* = 31)*
Country	
USA	30 (97%)
Canada^†^	1 (3%)
Specialty (some curricula included > 1 specialty)	
Internal medicine	6 (19%)
Family medicine	5 (16%)
Pediatrics	9 (29%)
Psychiatry^‡^	10 (32%)
Other specialty	5 (16%)
Teaching methods	
Experiential learning	19 (61%)
Small group discussion/seminar	15 (48%)
Lecture	20 (65%)
Independent project	6 (19%)
Group project	7 (23%)
Required reading	8 (26%)
Advocacy tools	
Legislative advocacy	18 (58%)
Community partnership/organizing	18 (58%)
Advocacy writing (op-ed, testimony, etc.)	13 (42%)
Public speaking	3 (10%)
Research-based advocacy	5 (16%)
Media relations	4 (13%)
Advocacy/policy content areas	
Social determinants of health	18 (58%)
Health equity / racial justice	11 (35%)
Healthcare finance	3 (10%)
Quality improvement	3 (10%)
Major health legislation (e.g., ACA)	4 (13%)
Structural competency	4 (13%)
Global health	4 (13%)
Evaluation	
Evaluation reported	21 (68%)
Content: trainee feedback/perceptions	21 (68%)
Content: trainee knowledge/skills/attitudes	14 (45%)
Survey	15 (48%)
Written feedback	3 (10%)
Focus group	4 (13%)
Interviews	4 (13%)
Stakeholder feedback	5 (16%)
Participant outcomes	4 (13%)

*n = 31 represents 31 overall curricula, extracted from 26 articles, with Vance (2020) and Kennedy (2018)—two articles that described the same 7 psychiatry advocacy curricula—counted as a total of 7 curricula

^†^Canadian article is Ying (2019)

^‡^Psychiatry total includes 7 curricula from Vance/Kennedy and 3 separate curricula from other articles

#### Teaching Methods

The most frequently used educational methods were experiential learning and lectures, followed by small group discussions. 77% of curricula (*n* = 24) described using multiple teaching methods, with 52% (*n* = 15) using both experiential learning and didactics (lecture, small group, or both). Projects were also a common tactic, with 38% (*n* = 24) of curricula utilizing individual and/or group projects. Only one curriculum reported using web-based modules^39^. Other unique educational methods described included panel debate^40^, writing an online blog^29^, developing advocacy alerts,^17^ and coaching.^11^

#### Skills-Based Advocacy Tools

Nearly all (94%, *n* = 29) of the curricula taught participants at least one advocacy tool, with 55% (*n* = 17) teaching more than one tool. Legislative advocacy skills, community partnership strategies, and advocacy writing (including op-eds, testimony, and other persuasive writing techniques) were most common. Some curricula utilized specific frameworks to teach specific tools (e.g., Asset-Based Community Development^42^ or Community-Based Participatory Research^39^ for community partnership) whereas others provided a broad overview of physician advocacy methods.^14^ Other unique tools included grant writing^18^ and legal research tools.^19^

#### Knowledge-Based Advocacy and Policy Content Areas

In addition to teaching tools needed to conduct advocacy work, 68% (*n* = 21) of curricula also covered specific topics relevant to advocacy and policy. Themes of inequity and social justice were prominent: SDOH and health equity/racial justice were the two most frequently taught topics, and four curricula taught structural competency. 60% (*n* = 19) of curricula included at least one of these three topics. Fewer curricula taught general health policy topics, with just 20% (*n* = 7) including quality improvement, healthcare finance, and/or major legislation (e.g., Affordable Care Act). Most curricula teaching legislative advocacy discussed local and state-level legislative pieces rather than national pieces of healthcare legislation. Several curricula included specific policy content on areas relevant to their specialties, such as mental health policy or child health policy topics (e.g., gun safety, SIDS, nutrition, etc), either alone or as part of broader themes on SDOH.

### Evaluation Methods

The considerable heterogeneity between the articles and the elements of curricular design and evaluation included in them limited our ability to evaluate the quality of each study using any standard scale. We did, however, assess articles on their evaluation techniques as one measure of quality. Each article was given a quality score based on the characteristics of their evaluation (A for multimodal evaluation, B for single method of evaluation, and C for no evaluation) (Table 4). A majority (68%, *n* = 21) of the articles reported results of a formal evaluation with many (42%, *n* = 13) engaging in multimodal evaluation methods. All reported evaluations focused on trainee perceptions and satisfaction, while 67% (*n* = 14) also reported on trainee knowledge and skill acquisition. Many of these evaluations were conducted via surveys (48%, *n* = 14), either pre- and post-curriculum surveys or post-curriculum surveys alone. A variety of qualitative methods, including focus groups and interviews, were also used to elicit feedback from participants and stakeholders such as community partners. Unique quantitative methods included differences in RVUs in a clinical setting,^10^ measures of blog success based on readership and media references to posts, and various measures of participant outcomes such as grant success, future degree acquisition, or advocacy career paths. Consideration for long-term impact and residents’ interest and likelihood to continue advocacy efforts beyond residency was given^7^ although not consistently measured.

**Table 4:** Evaluation Methods for Included Advocacy Curricula

First author, year		Evaluation reported	Knowledge/skills	Feedback/perceptions	Evaluation methods	Quality scale*
Andrews 2019		No				C
Bromage 2019		Yes	●	●	Focus group; stakeholder feedback	A
Campbell 2020		Yes	●	●	Written feedback; interview	A
Emery 2022		Yes		●	Survey; large group debrief	A
Gimpel 2017		Yes	●	●	Survey; participant outcomes	A
Goss 2020		Yes	●	●	Survey; participant outcomes	A
Hirsch 2017		Yes	●	●	Survey	B
Jones 2018		Yes		●	Survey; measures of blog success	A
Khera 2022		Yes		●	Survey	B
Knox 2018		Yes		●	Focus group; interview; stakeholder feedback	A
Krishnaswami 2018		Yes	●	●	Participant outcomes; 10-year evaluation plan with process and outcome measures	A
Lax 2019		Yes	●	●	Survey	B
Majeed 2020		Yes	●	●	Survey; written feedback	A
Michelson 2019		Yes	●	●	focus group; interview	A
Neff 2020		Yes	●	●	Written feedback	B
Oldfield 2018		No				C
Pak-Gorstein 2018		Yes	●	●	Survey; stakeholder feedback; participant outcomes; self-assessments	A
Piel 2018		No				C
Sieplinga 2021		Yes	●	●	Survey; stakeholder feedback; RVUs in clinic before/after curriculum	A
Teran 2020		Yes	●	●	Survey	B
Traba 2021		No				C
Vance 2020; Kennedy 2018	Harvard	Yes		●	Survey	B
	Hennepin	No				C
	UCSF	No				C
	U. Illinois	Yes		●	Survey	B
	U. Michigan	No				C
	UTSW	No				C
	Yale	No				C
Webber 2018		Yes		●	Survey; focus group; interview; stakeholder feedback	A
Whetstone 2018		No				C
Ying 2019		Yes	●	●	Survey	B

*Each curriculum was scored for its evaluation characteristics: A for multimodal evaluation, B for single method of evaluation, and C for no evaluation

### Core Elements of Curricular Design and Implementation

All included articles reflected upon lessons learned in curricular design and implementation based on evaluation results and the practical experience of leading advocacy curricula. We extracted recurrent themes meaningful for advocacy education.

#### Creating an Overarching Culture Supportive of Advocacy Education

Multiple articles highlighted the importance of GME programs creating buy-in for advocacy education by establishing a culture that supports advocacy training and efforts^14–16^^,^^19^^,^^21^^,^^26^. Early introduction of curricula^16^ was thought to promote advocacy-related knowledge acquisition, skill-building, and bonding^10^^,^^11^ and ultimately increase engagement in advocacy efforts throughout training.^17^ Some authors found that curricula should ideally be longitudinal with competency progression as a core component^20^^,^^26^.

Designation of resident and faculty champions^13^^,^^14^^,^^21^^,^^27^^,^^29^ was identified by several articles as a key strategy to promote curricular success and sustainability. Highlighting advocacy opportunities and outcomes at meetings, on program websites, and through listservs^21^^,^^26^ and providing platforms to share advocacy projects long-term^26^ were tools used to increase visibility and accessibility of advocacy-focused work, limit activation energy and encourage participation in this work^14^^,^^15^^,^^17^^,^^21^^,^^26^, reflect program identity to residency applicants during recruitment^7^^,^^18^^,^^21^^,^^26^^,^^31^, and promote longevity of efforts^26^.

Several publications discussed collaboration culture and reported that trainees benefit from working with other GME-level trainees^7^^,^^40^ across specialties^21^, participating in interdisciplinary^16^ and interprofessional^9^^,^^15^ teams, and engaging in systems-based learning^9^^,^^15^. Collaboration with the broader community, including other institutions and professional organizations^11^^,^^20^, was also helpful, especially when access to local advocacy resources was limited for various reasons, including geographic location^15^^,^^19^.

#### Designing Curricula to be Learner-Centric

Strategies to ensure that advocacy curricula are tailored to learner needs and interests were presented in several articles. Many emphasized the need for protected time to participate in offered curricula^12^^,^^15^^,^^17^^,^^19–21^^,^^27^^,^^42^, specifically recommending designated rotation blocks^7^^,^^10^^,^^18^^,^^26^^,^^41^, existing noon conferences^13^^,^^16^^,^^20^, academic half days^15^^,^^16^, and meals during meetings^7^^,^^11^^,^^12^^,^^21^ as strategies to facilitate curricular engagement. Recruitment of additional trainees to participate in advocacy endeavors was suggested to decreased burden of participation on trainees^18^.

Many authors proposed tactics to make advocacy education relevant and attainable to trainees. Several curricula recommended that educators explicitly discuss how developed skills can be applied in a medical career^9^^,^^12^^,^^14^ and emphasize that advocacy is achievable and important to physician identity irrespective of career path^13^. Establishing realistic and attainable goals, keeping the scope of projects feasible^42^, and allowing sufficient time for preparation of deliverables^42^ were methods used to prevent defeatism and burnout^12^. Clearly describing the objectives of curricular components and practical experiences upfront^26^^,^^32^^,^^42^, providing advance briefings^42^, and labeling advocacy activities as such^15^ enhanced trainees’ understanding of curricular purpose and intended impact.

In terms of outcomes, building and utilizing skill checklists^20^ were a suggested tool to track achievement. Scholarly opportunities were shown to be of importance to trainees^21^^,^^27^^,^^29^^,^^31^, and articles described ways to ensure trainees saw academic benefit from their advocacy education (e.g., residents interested in pursuing fellowship could combine advocacy efforts with research interests to produce scholarly work)^7^. Provision of academic recognition promoted a sense of achievement and career advancement^7^.

Articles described the importance of making curricula responsive to trainees. Explicitly recognizing trainee discomfort in engaging with advocacy education, creating supportive spaces for discussion and reflection^13^, and guiding learners through conversations regarding issues, challenges, and experiences in advocacy work^26^^,^^32^^,^^42^ were key themes. Authors recommended that educators discuss relevant facts in a manner that creates empathy for divergent viewpoints while dispelling incorrect preconceived notions and decreasing bias^40^. Providing feedback and tools to overcome barriers to successful advocacy was additionally supportive^14^.

Articles also described ways to make curricula adaptable to evolving learner needs^14^^,^^21^^,^^31^. Soliciting feedback from learners and then incorporating reported interests into curricular elements (e.g., selecting guest speakers—legislators, community activists, journalists, etc.—using trainee input)^15^^,^^21^, supporting trainee autonomy and ownership of advocacy work, and providing options (e.g., option to do projects individually or in small groups, option of topics for projects, etc) were strategies to customize the experience and make it more relevant^15^^,^^40^.

#### Supporting Educators

Educators trained in teaching advocacy are limited in number^14^^,^^16^^,^^18^^,^^19^^,^^21^, and several articles suggested that protected time for advocacy educators^14^^,^^19^ could facilitate curricular development, help existing instructors manage competing demands, and provide interested faculty time to gain relevant skills and experience^21^. Having multiple faculty facilitators for each curricular component also helped overcome logistical barriers associated with planning^20^, and some programs developed no- or low-cost^10^^,^^17^ curricular elements implementable without any faculty expertise.

Educators are expected to mentor trainees and role model constructive advocacy behaviors^14^, and a couple articles discussed strategies to support this role. One article recommended utilizing peer mentorship, group mentorship, and mentors outside the institution if advocacy champions are not immediately available^14^; another article recommended that programs provide clear expectations for faculty members regarding their specific mentorship responsibilities^26^.

#### Including Action-Oriented Components

Advocacy is a skills-based pursuit^14^, and trainees value real-world application of advocacy frameworks^9^^,^^10^^,^^12^^,^^14^ as well as active learning opportunities^40^. Thus, multiple articles suggested that educational efforts should focus on action and practical aspects of advocacy^40^ rather than discussions surrounding theoretical advocacy and political viewpoints^14^^,^^40^. Incorporation of action-oriented and timely topics was suggested as a potential strategy to improve attendance especially when residents are on busy rotations and less motivated to engage^7^.

Trainees often consider advocacy to be primarily patient-centered and individual-focused^12^^,^^14^^,^^15^. Several articles suggested integrating advocacy work with existing clinical responsibilities^10^ to make the impact of advocacy more visible^12^, bridge the gap between recognizing an issue and engaging in work that decreases disparities^16^^,^^26^, and support trainees in guiding tangible improvements in their clinical practice^13^^,^^15^^,^^26^. Taking these efforts a step further, community service opportunities may allow physicians to interact on a human level with their patients, adjusting the power dynamic that often exists in the clinical setting^15^ and moving trainees beyond a purely individual concept of advocacy. Multiple articles cautioned that advocacy activities involving patients and communities should adapt to their evolving needs^12–16^, engage nonclinical and community stakeholders^28^^,^^31^^,^^42^, minimize burden for community partners^42^, and demonstrate community impact^18^. Efforts should be sustainable for the community involved as well^33^. Care should be taken that efforts are patient-relevant and not just serving to address physician interests^14^.

Several curricula incorporated action-oriented activities beyond the community-level, most commonly utilizing field trips to state capitols as an experiential strategy to help learners practice persuasive legislative advocacy skills^14^^,^^19^^,^^21^; this strategy presented challenges though when instruction timing did not align with legislative windows^19^. Project-focused work potentially led to better outcomes such as increased camaraderie, leadership opportunities, and effectiveness^7^^,^^40^, but connecting trainees with longitudinal projects was sometimes time-consuming and may benefit from utilizing national networks over local ones moving forward^7^.

## DISCUSSION

Advocacy is a core part of physician training that requires active instruction; it is no longer sufficient to assume trainees will acquire essential advocacy knowledge and skills through just passive exposure^21^. Authors of prior publications have sought to determine components of an ideal curriculum for advocacy education^14^^,^^34^^,^^43–45^, and our work builds upon this. Similar to Howell et al., we found that GME advocacy curricula most frequently focus on grassroots advocacy and community partnership, legislative advocacy, and persuasive communication in terms of topics covered, and most utilize lecture and experiential learning elements for teaching methods. While more curricula reviewed by Howell et al. included projects, our review showed that more recently published curricula instead include small group sessions. Specialties most represented in both reviews were Internal Medicine, Pediatrics, and Family Medicine while our updated review had substantial representation of curricula in Psychiatry as well. Both reviews noted heterogeneity in curricular evaluation methods while we further investigated the nature of the variability. Beyond the work of Howell et al., we found that content areas of most interest include social and structural determinants of health as well as health equity and racial justice. Our work also revealed additional insights regarding core features and components of GME advocacy curricula, building upon the work of Vance et al. (included in our review) who identified core components (didactics and experiential learning), attributes (practical, adaptive, patient-focused), and supports (champions, buy-in, and mentorship) across curricula in Psychiatry. Our common findings with these prior publications support the notion of an emerging consensus on core elements of advocacy curricula while our updated findings may reflect a shift in current advocacy teaching trends at the GME level.

**Figure 2:**
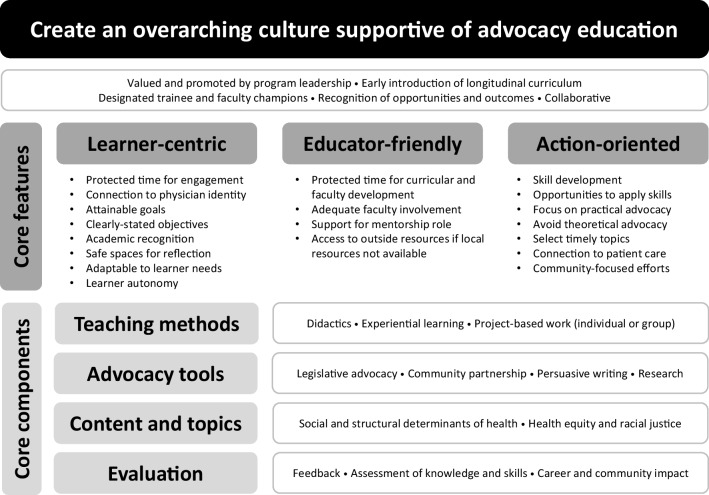
Proposed framework for design and implementation of GME advocacy education curricula.

We have combined our findings with the results of prior publications to propose an innovative and integrative framework grounded in experience that can be used to support and sustain advocacy education for GME trainees (Fig. 2). We specifically propose that programs should create culture that supports advocacy curricula, recognizes the unique needs of learners and educators, focuses on action-oriented skill development, utilizes a variety of teaching methods and advocacy tools, incorporates content and topics of relevance and interest, and conducts evaluation of impact on trainees and communities. The elements of our framework importantly align with several ACGME core requirements for residents and fellows^23^^,^^24^ (Table 5) as well as Malcolm Knowles’s Adult Learning Theory.

**Table 5:** Alignment of Proposed Framework with ACGME Core Requirements for Residents and Fellows

ACGME section	Residency requirement	Fellowship requirement	Description of requirement	Corresponding framework component
IV.A.2	●	●	*Competency-based goals and objectives for each educational experience designed to promote progress on a trajectory to autonomous practice*	Early introduction of advocacy curriculum with some support for competency progression
IV.A.4.a)	●		*Residents must be provided with protected time to participate in core didactic activities*	Learner-centric with protected time for trainee engagement
IV.B.1.f).(1).(c)	●		*Advocating for quality patient care and optimal patient care systems*	Action-oriented with connection to patient care
IV.D.1.b)	●	●	*The program, in partnership with its Sponsoring Institution, must allocate adequate resources to facilitate resident/fellow and faculty involvement in scholarly activities*	Learner-centric and educator-friendly curriculum with protected time for both
VI.C.1.a)	●	●	*Efforts to enhance the meaning that each resident/fellow finds in the experience of being a physician, including […] promoting progressive autonomy and flexibility*	Learner-centric with connection to physician identity
VI.C.1.e)	●	●	*Attention to resident/fellow and faculty member burnout*	Learner-centric with connection to physician identity
VI.E.2	●	●	*Opportunity to work as a member of effective interprofessional teams*	Overarching culture supportive of collaboration
VI.A.1.b).(1).(a)	●	●	*Residents/fellows must receive training and experience in […] understanding of healthcare disparities*	Core content and topics
II.A.4.a).(2) [Background and Intent]	●	●	*The mission of institutions participating in graduate medical education is to improve the health of the public. Each community has health needs that vary based on location and demographics. Programs must understand the social determinants of health of the populations they serve and incorporate them in the design and implementation of the program curriculum, with the ultimate goal of addressing these needs and health disparities*	Action-oriented with inclusion of community-focused efforts; core content and topics; evaluation of community impact

An overarching GME culture supportive of advocacy education and efforts is foundational to building curricula that are sustainable longitudinally. Trainee engagement in curricular elements requires time, and creation and delivery of curricular content are highly time-intensive. We support the call of multiple authors that programs should provide protected time for both trainees and educators. Skilled instructors are limited and should be considered a valuable asset.

Multiple curricula explicitly state the need for combining didactics with experiential learning, but we propose that the addition of action-oriented project-based work, whether completed individually or in groups, is also important. Most curricula include didactics, which can be incorporated into existing lecture/conference time to provide learners with protected time. Experiential learning opportunities can be incorporated with clinical duties to increase trainee connection with clinical work. Project-based work and community collaboration often go together although this is not necessary. Only one curriculum we reviewed utilized web-based modules although, since the COVID-19 pandemic, this is likely not representative of the current state of advocacy education.

Beyond op-ed writing, writing tools in general are considered important. Media engagement strategies, such as social media posts, overlap with writing skills. Many curricula and associated projects depend on funding from various grants^7^^,^^11^^,^^13^^,^^20^^,^^27^^,^^28^^,^^42^, and it is likely beneficial to specifically include grant-writing skills in advocacy curricula^18^.

In terms of content, health disparities in marginalized populations are of frequent interest^28^^,^^32^. Curricula should likely also teach advocacy ethics^19^ although this was only highlighted in a few articles.

Evaluation is generally lacking; most curricula utilize surveys to elicit trainee feedback and perceptions rather than objectively measure attainment of knowledge and skills. This area can use focus as advocacy is an action-oriented, skills-based pursuit. We recommend educators utilize Levels 3 and 4 of the Kirkpatrick Model of Evaluation to collect data during and after GME training to gain insight into the usefulness and utilization of advocacy skills learned in training^11^^,^^13^^,^^18^^,^^20^^,^^26^^,^^29^^,^^31^ and the impact of training on patient and community health^8^^,^^31^.

Our proposed framework is a starting point that we hope will generate a broad structure for advocacy curricula that can be adapted by individual programs. A future consensus process to affirm and finalize this structure is necessary as the degree to which our findings represent a complete landscape of GME advocacy curricula is limited by several factors. Our search extracted only published curricula, meaning valuable information from unpublished curricula was not available for analysis. There were likely also articles that discussed advocacy tools and content without labeling them as such and were thus not captured in our search despite our attempts to use inclusive search terms. Of the curricula we did capture, some articles lacked significant details, so the actual curricula may have included methods, tools, and content areas not reflected in our analysis. Most articles discussed a single curriculum at one institution, so results may not be generalizable although we attempted to address this by primarily extracting themes discussed across multiple publications.

Although our data represents a majority of Psychiatry curricula given the work of Vance et al. and Kennedy et al., Pediatrics is often regarded as having the most well-developed advocacy curricula, which may be secondary to the ACGME’s explicit requirement for pediatric trainees to learn about advocacy. In the future, explicit recognition of advocacy education as essential by the ACGME can enhance the presence and efficacy of advocacy curricula across specialties^21^. Resources and support from national organizations can also be helpful^21^. While our work adds to prior studies to determine effective teaching methods, core advocacy tools, and important content areas, further work is necessary to make sure these findings are truly representative of advocacy curricula being developed and taught across GME.

## CONCLUSION

Modern physicians must demonstrate proficiency in advocacy skills to meaningfully care for their patients and patient populations; incorporating formal, comprehensive advocacy curricula into GME programs can facilitate competency achievement. Our findings from a systematic review of recently published GME advocacy curricula, combined with findings from previously published curricula, reveal recurrent core components that can be used to develop foundational advocacy education modules for dissemination and adaptation across GME programs. These include building program culture supportive of advocacy education; designing curricula to be action-oriented and mindful of learner and educator needs; utilizing didactics, experiential learning, and projects to teach advocacy; covering advocacy tools including legislative advocacy, community partnership, persuasive writing, and research methods; and teaching social and structural determinants of health, health equity, and racial justice. Next steps are to build expert consensus on core features and components of advocacy curricula and utilize our proposed framework to design standardized advocacy modules and mitigate some of the repetitive burden that educators face in teaching advocacy.

## Supplementary Information

Below is the link to the electronic supplementary material.

Supplementary file1 (DOCX 17 KB)
