# Supplementary material for: Advocacy Curricula in Graduate Medical Education: an Updated Systematic Review from 2017 to 2022
Source: J Gen Intern Med. 2023 Jun 20;38(12):2792–807. doi: 10.1007/s11606-023-08244-x (PMC10507002; doi:10.1007/s11606-023-08244-x)
Supplement: Supplementary file 1 — Supplementary file1 (DOCX 17 KB) [file 11606_2023_8244_MOESM1_ESM.docx]

**Appendix.** Systematic database search strategies

**PubMed**

(“education, medical, graduate”[MESH] OR “internship and residency”[MESH] OR (medical education[tiab] and (physician’s role[tiab] or residen*[tiab]))) AND (curriculum[MESH] OR curricul*[tiab]) AND (Advoca*[tiab] OR “Child Advocacy”[Mesh] OR “Patient Advocacy”[Mesh] OR “community engagement”[tiab] OR (communit*[tiab] AND engag*[tiab]) OR “community-based participatory research”[MESH] OR “community-institutional relations”[MESH] OR canmed[tiab] OR canmeds[tiab] OR “Community Medicine/education”[Mesh] OR “human rights”[MESH] OR “social justice”[MESH] OR “lobbying”[MESH] OR “vulnerable populations”[MESH] OR “poverty”[MESH]) AND 2017/09/01:3000[dp] NOT (“Africa”[Mesh] OR “Asia”[Mesh] OR “Europe”[Mesh] OR “Oceania”[Mesh])

**Embase** (embase.com)

(‘graduate medical education’/exp OR ‘residency education’/exp OR ‘graduate medical education’:ab,ti OR ‘resident education’:ab,ti OR ‘residency education’:ab,ti ) AND (‘curriculum development’/exp OR ‘curriculum’/exp OR curricul*:ab,ti) AND (‘child advocacy’/exp OR ‘patient advocacy’/exp OR advoca*:ab,ti OR ‘lobbying’/exp OR lobbying:ab,ti OR (community NEAR/3 relations):ab,ti OR ‘community assessment’/exp OR ‘community assessment’:ab,ti OR ‘public health’/exp OR ‘health care system’/exp OR ‘human rights’/exp OR ‘community engagement’:ab,ti OR (engage* NEAR/3 communit*):ab,ti OR ‘community integration’/exp OR ‘community’/exp OR ‘community assessment’/exp OR community:ti,ab OR ‘community dynamics’/exp OR ‘community ecology’/exp) AND [english]/lim AND [01-09-2017]/sd NOT [05-03-2022]/sd

**PsycINFO (via ProQuest)**

(MAINSUBJECT.EXACT(“Medical Residency”) OR MAINSUBJECT.EXACT(“Medical Internship”) OR MAINSUBJECT.EXACT(“Medical Education”)) AND (MAINSUBJECT.EXACT(“Curriculum”)) AND (MAINSUBJECT.EXACT(“Community Involvement”) OR MAINSUBJECT.EXACT(“Advertising”) OR MAINSUBJECT.EXACT(“Advocacy”) OR tiab(“community engagement” OR “advocacy education” OR “advocacy training”) OR tiab(engage* N/3 communit*))

Limited to publications from September 1, 2017, forward.

**ERIC (via Proquest)**

(MAINSUBJECT.EXACT(“Medical Education”) OR MAINSUBJECT.EXACT(“Graduate Medical Education”)) AND (MAINSUBJECT.EXACT(“Curriculum”) OR MAINSUBJECT.EXACT(“Courses”) OR MAINSUBJECT.EXACT(“Course Content”)) AND (MAINSUBJECT.EXACT(“Advocacy”) OR MAINSUBJECT.EXACT(“Child Advocacy”) OR MAINSUBJECT.EXACT(“Community Involvement”) OR AB,TI(“community engagement” OR “advocacy education” OR “advocacy training”) OR AB,TI(engage* N/3 communit*))

*Searched without date limit because original strategy unknown*

**MedEdPORTAL ( www.mededportal.org)**

Manual search for all articles from 2017-present, for the terms ADVOCA*, “Community Engagement,” “Community Based Participatory Research,” “Community Institutional Relations,” “Community Medicine,” “Human Rights,” “Social Justice,” Lobbying, “Vulnerable Populations,” Poverty. Manual de-duplication and removal of articles from Jan–Aug 2017.
